# Risk Stratification Model for Predicting Coronary Care Unit Readmission

**DOI:** 10.3389/fcvm.2022.825181

**Published:** 2022-02-24

**Authors:** Tien-Yu Chen, Chien-Hao Tseng, Po-Jui Wu, Wen-Jung Chung, Chien-Ho Lee, Chia-Chen Wu, Cheng-I Cheng

**Affiliations:** ^1^Division of Cardiology, Department of Internal Medicine, Kaohsiung Chang Gung Memorial Hospital, Kaohsiung, Taiwan; ^2^Division of Cardiothoracic and Vascular Surgery, Department of Surgery, Chang Gung Memorial Hospital Kaohsiung Branch, Kaohsiung, Taiwan; ^3^School of Medicine, College of Medicine, Chang Gung University, Taoyuan, Taiwan

**Keywords:** coronary care unit (CCU), readmission, prediction score model, discharge, risk

## Abstract

**Background:**

Use of statistical models for assessing the clinical risk of readmission to medical and surgical intensive care units is well established. However, models for predicting risk of coronary care unit (CCU) readmission are rarely reported. Therefore, this study investigated the characteristics and outcomes of patients readmitted to CCU to identify risk factors for CCU readmission and to establish a scoring system for identifying patients at high risk for CCU readmission.

**Methods:**

Medical data were collected for 27,841 patients with a history of readmission to the CCU of a single multi-center healthcare provider in Taiwan during 2001-2019. Characteristics and outcomes were compared between a readmission group and a non-readmission group. Data were segmented at a 9:1 ratio for model building and validation.

**Results:**

The number of patients with a CCU readmission history after transfer to a standard care ward was 1,790 (6.4%). The eleven factors that had the strongest associations with CCU readmission were used to develop and validate a CCU readmission risk scoring and prediction model. When the model was used to predict CCU readmission, the receiver-operating curve characteristic was 0.7038 for risk score model group and 0.7181 for the validation group. A CCU readmission risk score was assigned to each patient. The patients were then stratified by risk score into low risk (0–12), moderate risk (13–31) and high risk (32–40) cohorts check scores, which showed that CCU readmission risk significantly differed among the three groups.

**Conclusions:**

This study developed a model for estimating CCU readmission risk. By using the proposed model, clinicians can improve CCU patient outcomes and medical care quality.

## Background

An intensive care unit (ICU) readmission is associated with undesirable outcomes, including increases in mortality, duration of hospital stay, and medical costs (1, 2).

ICU readmissions is an essential consideration for three reasons. First, readmissions consume substantial financial and medical resources, regardless of whether the patient is readmitted for the same illness or an unrelated illness. Second, ICU readmissions is often used as a convenient indicator of care quality. Third, reported ICU readmission rates remain high, ranging from 4 to 14% (2). When determining whether a patient is ready for discharge, ICU personnel often rely solely on their own clinical experience and judgment. Thus, the decision is usually highly subjective (3). Insufficient hospital beds may result in premature discharge of ICU patients, some of whom inevitably require readmission. Therefore, identifying patients with a high risk of ICU readmission would not only prevent discharges of patients who are not ready for transfer to standard care, it would also reduce morbidity and mortality after discharge.

Many previous studies have addressed this problem by investigating risk factors for ICU readmission. Thus, various risk factors have been identified, and many different solutions have been proposed for estimating and reducing ICU readmission risk (4–8). However, no studies have validated a scoring system for predicting coronary care unit (CCU) readmission risk. Clinical characteristics substantially differ between CCU and ICU patients. For example, CCU patients typically receive care for an acute episode of congestive heart failure or acute myocardial infarction whereas medical ICU patients typically receive care for sepsis or acute respiratory failure.

The objectives of this study were to identify characteristics and outcomes in patients readmitted to CCU in order to identify factors that increase CCU readmission risk and to establish a scoring system for predicting patients who have a high risk of CCU readmission.

## Materials and Methods

### Data Source

This retrospective observational study analyzed data from the Chang Gung Research Database (CGRD) provided by the Chang Gung Medical Foundation, Taiwan. The CGRD contains complete electronic medical records (EMR) data from seven Chang Gung Memorial Hospital branches in seven different counties/cities in Taiwan, including three medical centers and four regional/district hospitals, which have 10,050 total beds (9, 10). The CGRD, which is the largest EMR database in Taiwan, contains records for approximately 6.1% of outpatients and 10.2% of inpatients in Taiwan (9, 10).

The identification number of each patient in the CGRD is encrypted and de-identified to protect privacy. Therefore, informed consent was waived for this study. The diagnosis and laboratory data could be linked and continuously monitored using consistent data encryption. This study was approved by the Institutional Review Committee on Human Research at our institution (IRB: 201900829B0C501). This study complied with the Declaration of Helsinki. The interpretation and conclusions contained herein do not represent the views of Chang Gung Memorial Hospital.

### Study Patients and Setting

**Figure 1:**
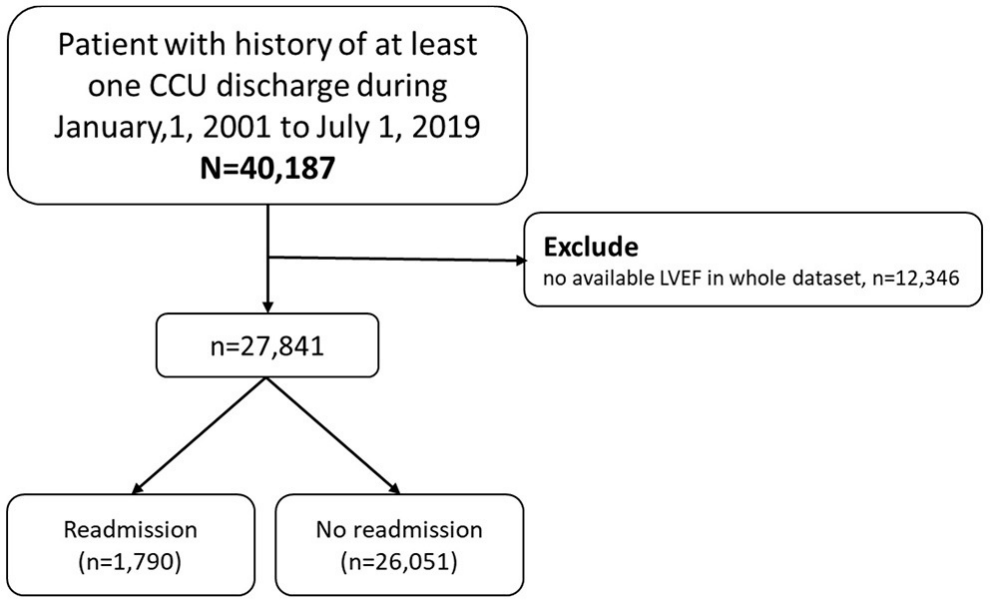
Study design and screening criteria flow chart. CCU, coronary care unit.

The CGRD search included EMRs for patients treated January 1, 2001, to July 1, 2019. Data were retrieved for all patients with a history of at least one CCU discharge (*N* = 40,187). After excluding patients without left ventricle ejection fraction (LVEF) data, the final number of participants in model building was 27,841. The flowchart of the study design and patient enrollment is shown in Figure 1. These patients were then separated into readmission and non-readmission groups, where readmission was defined as readmission to CCU after initial CCU discharge and non-readmission was defined as absence of CCU readmission history after initial CCU discharge, death after CCU discharge, or transfer from CCU to general ward.

This study was performed in two steps. First, the study data were segmented at a ratio of 9 to 1 for model building and model validation, respectively. That is, 90% of data were used to develop the prediction model, including variable selection, scoring, and model building. The remaining 10% of data were then used to validate the developed prediction model. The capability of the model was evaluated in terms of specificity, sensitivity, and positive predictive value.

### Measurements

The primary outcome of interest was defined as any CCU readmission after CCU discharge from the index admission. Secondary outcomes of interest were CCU length of stay, in-hospital mortality and hospital costs during admission. Criteria for CCU admission included, but were not limited to, (1) a need for mechanical ventilation or airway protection, invasive hemodynamic monitoring, or circulatory assistive device such as extracorporeal membrane oxygenation (ECMO) or intra-aortic balloon pump (IABP), (2) medication requiring continuous hemodynamic monitoring, and (3) acute myocardial infarction, acute coronary syndrome, or post-intervention procedure such as left main stent placement or carotid artery stenting.

Covariate analysis included demographic, physiological and laboratory data on the day of discharge. Specifically, the analysis included age, gender, body weight, and smoking history. Underlying comorbidities included in the covariate analysis were hyperlipidemia, congestive heart failure (CHF), stroke, peripheral artery occlusive disease, atrial fibrillation, chronic kidney disease (eGFR <60), end stage renal disease under dialysis, diabetes mellitus, hypertension, coronary artery disease, chronic obstructive pulmonary disease, liver cirrhosis, percutaneous coronary intervention, myocardial infarction, coronary artery bypass graft (CABG) and Charlson comorbidity index. Since using continuous variables increases the complexity of calculations when establishing the risk score model, we convert the selected continuous variables into categorical variables according to clinical practice or references. Covariate analysis also included cause of ICU admission; source of initial ICU admission (emergency department vs. general ward); treatment with ventilator, ECMO or IABP during CCU stay; sepsis during CCU stay (pneumonia, urinary tract infection, intraabdominal infection or other bacteremia); and bleeding during CCU stay (intracranial bleeding, intra-abdominal bleeding, gastrointestinal bleeding and other bleeding).

### Statistical Analysis

Data were presented as means and standard deviations for continuous variables and as proportions for categorical variables. Analysis of variance was performed to analyze differences in continuous variables. The χ^2^ test was used to analyze differences in nominal variables. To identify independent predictors of CCU readmission, explanatory covariates with a *p* < 0.01 in univariate analysis were entered into a multivariable regression model in a forward/backward fashion. In order to facilitating clinical application, we eliminated variables which are less contributory based on literature, clinical knowledge and completeness of the data to assemble an easy model to assist clinicians. The results of the multivariable regression analysis were then used to derive the clinical prediction model with CCU readmission as the outcome variable. Point values were assigned according to a rough multiple of the odds ratio (OR) for each variable. An area under the receiver operator curve (AUROC) was drawn to estimate the accuracy of the model in predicting CCU readmission risk at the time of CCU discharge. Statistical significance was defined as a *P* < 0.05. All analyses were performed using SAS 9.2 (SAS Institute Inc., Cary, NC, USA).

## Results

From January 1, 2001 to July 1, 2019, 27,841 patients were discharged from our CCU. Of these, 1,790(6.4%) were “bounce back” patients, i.e., patients readmitted to CCU after discharge to a general ward.

Table 1 presents the characteristics of the groups at the time of admission, i.e., before transfer to a ward. Patient characteristics statistically associated with readmission included older age, female gender, low body weight, no tobacco use, and any history of the following: hyperlipidemia, CHF, stroke, peripheral artery occlusive disease, atrial fibrillation, chronic kidney disease stage>3, dialysis, hypertension, chronic lung disease, diabetes, chronic liver disease, percutaneous coronary intervention, myocardial infarction, and CABG.

**Table 1:** Baseline characteristics.

	**Readmission** ***N*** **= 1,790**	**No readmisison** ***N*** **= 26,051**	***P* value**
Age	73.08 ± 12.35	67.73 ± 72.03	<0.0001
Male	972 (54.30)	16,324 (62.66)	<0.0001
Body weight (kg)	61.94 ± 28.11	63.85 ± 14.86	0.0143
Smoking	198 (11.06)	4,312 (16.55)	<0.0001
Prior hyperlipidemia	845 (47.21)	9,113 (34.98)	<0.0001
Prior CHF	764 (42.68)	5,523 (21.2)	<0.0001
Prior stroke	431 (24.08)	3,905 (14.99)	<0.0001
Prior PAOD	123 (6.87)	815 (3.13)	<0.0001
Prior AF	347 (19.39)	3,103 (11.91)	<0.0001
CKD			<0.0001
eGFR >60 stage1,2	525 (29.33)	13,115 (50.34)	
eGFR <60 stage3,4,5	1,265 (70.67)	12,936 (49.66)	
Prior dialysis	286 (15.98)	2,308 (8.86)	<0.0001
Prior hypertension	1,297 (72.46)	14,552 (55.86)	<0.0001
Prior COPD	449 (25.08)	4,508 (17.3)	<0.0001
Prior DM	969 (54.13)	9,266 (35.57)	<0.0001
Prior liver disease	342 (19.11)	4,191 (16.09)	<0.0001
Prior PCI	205 (11.45)	1,238 (4.75)	<0.0001
Prior MI	571 (31.9)	3,688 (14.16)	<0.0001
Prior CABG	49 (2.74)	345 (1.32)	<0.0001
**SBP before transfer**			
Systolic (mmHg)	135.5 ± 27.87	137.2 ± 25.84	0.0375
Diastolic (mmHg)	71.59 ± 14.72	74.56 ± 14.59	<0.0001
HR before transfer (g/dl)	81.91 ± 18.89	81.30 ± 18.23	0.2605
Ventilator during CCU	304 (16.98)	3,468 (13.31)	<0.0001
IABP during CCU	106 (5.92)	2,093 (8.03)	0.0013
ECMO during CCU	17 (0.95)	888 (3.41)	<0.0001
OHCA before admission	25 (1.4)	197 (0.76)	0.0032
IHCA before admission	79 (4.41)	1,493 (5.73)	0.0195
Sepsis during CCU	745 (41.62)	6,888 (26.44)	<0.0001
Bleeding during CCU	252 (14.08)	2,790 (10.71)	<0.0001
ACS before admission	679 (37.93)	14,679 (56.35)	<0.0001
Cardiac arrest during CCU	54 (3.02)	819 (3.14)	0.7654
**Lab data before transfer**			
Hb (g/dl)	10.76 ± 2.09	11.78 ± 2.54	<0.0001
Hct (%)	32.79 ± 6.20	35.43 ± 7.09	<0.0001
WBC (10^4^/L)	10.92 ± 4.83	10.41 ± 5.11	<0.0001
Platelet (10^9^/L)	201.6 ± 98.82	201.4 ± 92.94	0.9444
INR	1.19 ± 0.44	1.17 ± 0.48	0.0258
Cre (mg/dL)	2.84 ± 2.60	2.23 ± 2.54	<0.0001
eGFR (ml/min/1.73m^2^)	45.41 ± 39.74	62.05 ± 45.14	<0.0001
Na	139.0 ± 7.45	138.8 ± 7.97	0.2048
K	4.11 ± 2.20	4.06 ± 1.34	0.2772
LVEF before transfer	51.74 ± 18.12	56.00 ± 17.25	<0.0001
In-hospital mortality	446 (24.92)	3,093 (11.87)	<0.0001
Charlson comorbidity index	3.43 ± 3.10	1.94 ± 2.54	<0.0001
Readmission duration since transfer (day)	9.22 ± 8.46		
Cost (TWD)	433,217 ± 267,890	212,782 ± 220,212	<0.0001
CCU stay length	17.11 ± 20.16	6.51 ± 10.23	<0.0001
Transfer at weekend	246 (13.74)	4,434 (17.02)	<0.0001
Route of CCU admission			<0.0001
ED	1,667 (93.13)	23,340 (89.59)	
Ward	123 (6.87)	2,711 (10.41)	

*Data are expressed as mean ± standard deviation or as numbers (percentage)*.

*CHF, congestive heart failure; PAOD, peripheral artery occlusive disease; AF, atrial fibrillation; CKD, chronic kidney disease; COPD, chronic obstructive pulmonary disease; DM, diabetes mellitus; PCI, percutaneous coronary intervention; MI, myocardial infarction; CABG, coronary artery bypass graft surgery; SBP, systolic blood pressure; HR, heart rate; IABP, intra-aortic balloon pumping; ECMO, extracorporeal membrane oxygenation; OHCA, out-of-hospital cardiac arrest; IHCA, in-hospital cardiac arrest; ACS, acute coronary syndrome; Hb, hemoglobin; HCT, hematocrit; WBC, white blood cell; INR, international normalized ratio; Cre, creatinine; LVEF, left ventricular ejection fraction; CCU, coronary care unit; ED, emergency department*.

Compared to non-readmission patients, readmission patients also had lower diastolic blood pressure before transfer and a higher out-of-hospital cardiac arrest rate before CCU readmission. However, the readmission patients had a lower in-hospital cardiac arrest rate before the first CCU admission compared to the non-readmission patients. During their first CCU stay, readmission patients also tended to have lower rates of IABP use and ECMO use but a higher rate of ventilator use compared to the non-readmission patients. Readmission patients also had higher rates of sepsis and bleeding. However, readmission patients had lower hemoglobin, lower glomerular filtration rate, and lower left ventricle ejection fraction. By basing on clinical practice where LVEF <30% is defined as severe heart failure and several other parameters we analyzed, we had chosen age>80 and LVEF <30% as our threshold to obtain the best predictability. Finally, readmission patients had higher Charlson comorbidity index, higher in-hospital mortality, higher medical costs, and longer CCU stays compared to non-readmission patients.

After univariate and step-wise multivariate logistic regression analysis, factors that revealed positive associations with CCU readmission (Table 2) were history of CHF [OR 1.794, 95% confidence interval (CI)1.601–2.01], history of peripheral artery occlusive disease [OR 1.328, 95% CI 1.067–1.654], history of chronic kidney disease (eGFR <60) [OR 1.689, 95% CI 1.503–1.899], history of diabetes mellitus [OR 1.427, 95% CI 1.28–1.592], history of percutaneous coronary intervention [OR 1.4, 95% CI 1.16–1.689], history of myocardial infarction [OR 1.776, 95% CI 1.558–2.025], sepsis during CCU stay [OR 1.608, 95% CI 1.443–1.791], bleeding during CCU stay [OR 1.184, 95% CI 1.019–1.376], Admission to CCU from emergency department [OR 1.359, 95% CI 1.113–1.661], Age>80 [OR 1.249, 95% CI 1.115–1.398], LVEF <30% [OR 1.329, 95% CI 1.134–1.557]

**Table 2:** Multivariable analysis for readmission.

	***P*-value**	**Odds ratio**	**95%CI**	
Prior CHF	<0.0001	1.794	1.601	2.010
Prior PAOD	0.0112	1.328	1.067	1.654
Prior CKD	<0.0001	1.689	1.503	1.899
Prior DM	<0.0001	1.427	1.280	1.592
Prior PCI	0.0004	1.400	1.160	1.689
Prior MI	<0.0001	1.776	1.558	2.025
Sepsis during CCU	<0.0001	1.608	1.443	1.791
Bleeding during CCU	0.0277	1.184	1.019	1.376
CCU admission from ER	0.0027	1.359	1.113	1.661
Age > 80y	0.0001	1.249	1.115	1.398
LVEF <30%	0.0004	1.329	1.134	1.557

*CHF, congestive heart failure; PAOD, peripheral artery occlusive disease; CKD, chronic kidney disease; DM, diabetes mellitus; PCI, percutaneous coronary intervention; MI, myocardial infarction; ER, emergency department*.

**Figure 2:**
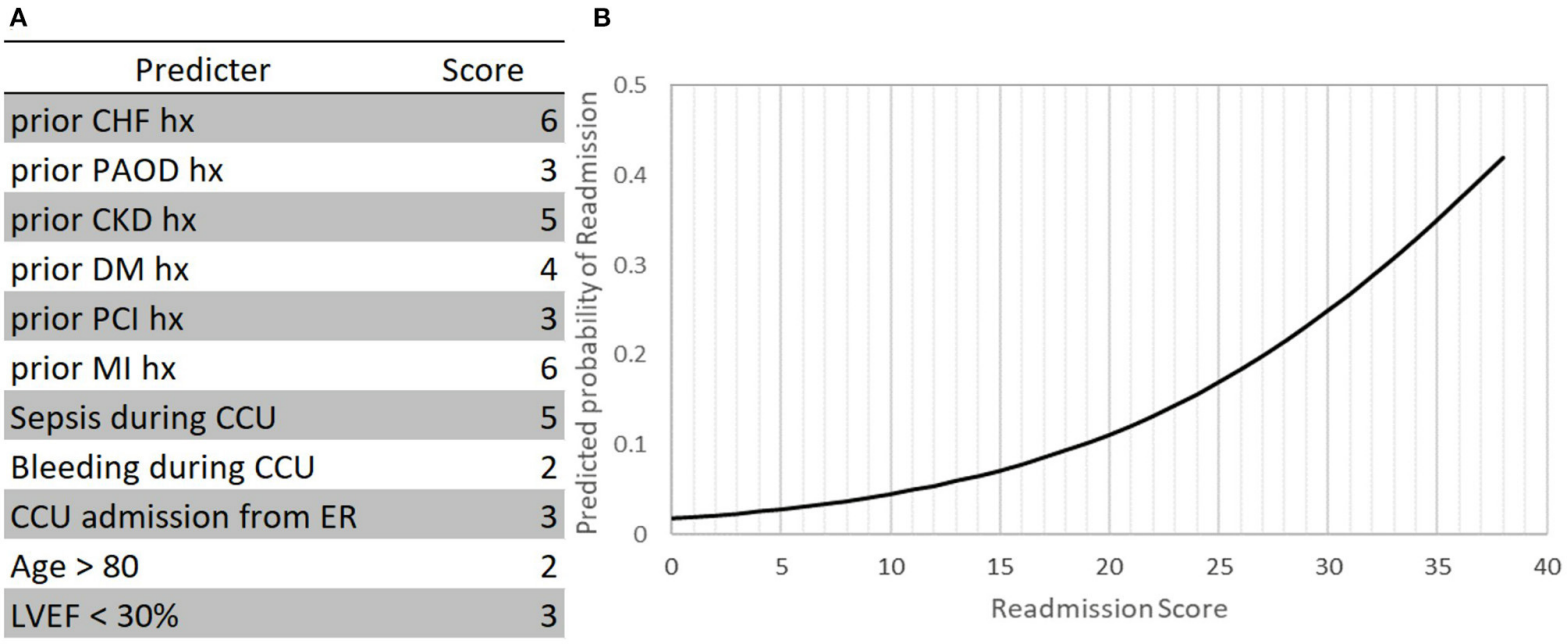
CCU readmission risk score model nomogram. **(A)** The CCU readmission risk score and **(B)** mean predicted CCU readmission by CCU readmission risk score. CCU, coronary care unit; CHF, congestive heart failure; PAOD, peripheral artery occlusive disease; CKD, chronic kidney disease; DM, diabetes mellitus; PCI, percutaneous coronary intervention; MI, myocardial infarction; LVEF, left ventricular ejection fraction.

In the multivariable model, variables that were independently associated with CCU readmission risk score were obtained from 90% of the study population (Figure 2A). Scores for CCU readmission risk were assigned to the aforementioned variables based on their relative ORs. Figure 2B shows the relationship between risk score and predicted probability of CCU readmission. Notably, the figure shows that the rise in risk score corresponded with the rise in the predicted risk of CCU readmission. The patients were then stratified by readmission risk into three cohorts: a low risk cohort (0–12), a moderate risk cohort (13–31), and a high-risk cohort (32–40). Table 3 further shows that the predicted risk of readmission was 0–5%, 5–30%, and > 30% in the low-, moderate- and high-risk cohorts, respectively. The predicted readmission rate by risk-cohort in validation cohort is in Supplement Table 1.

**Table 3:** Predicted readmission rate by risk-cohort (over all).

**Risk score cohorts**	**Score total**	**Predicted risk of readmission**	**% of cohort (*n* = 27,841)**
Low risk of readmission	0–12	0–5%	16,389 (58.87%)
Moderate risk of readmission	13–31	5–30%	11,249 (40.4%)
High risk of readmission	32–40	>30%	203 (0.73%)

**Figure 3:**
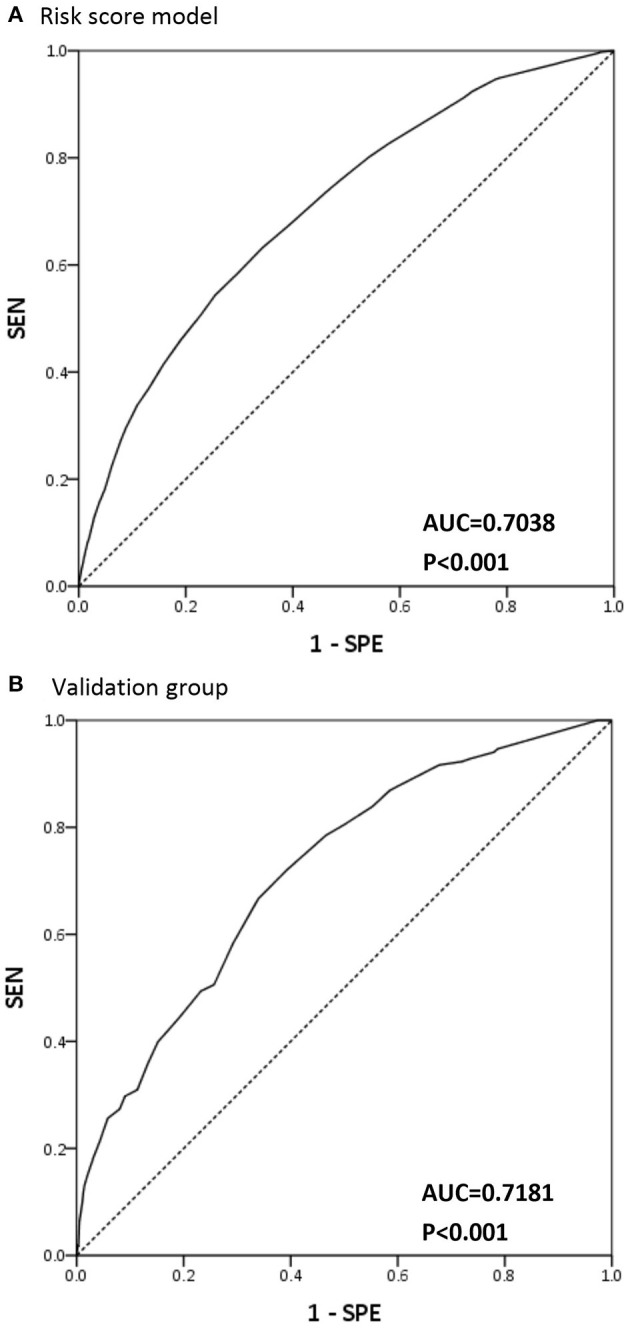
AUC curve for readmission risk score model and validation group. **(A)** Area under the receiver operator curve (AUC) for risk score model in predict coronary care unit readmission. **(B)** AUC for risk score model in predict coronary care unit readmission in validation group.

**Figure 4:**
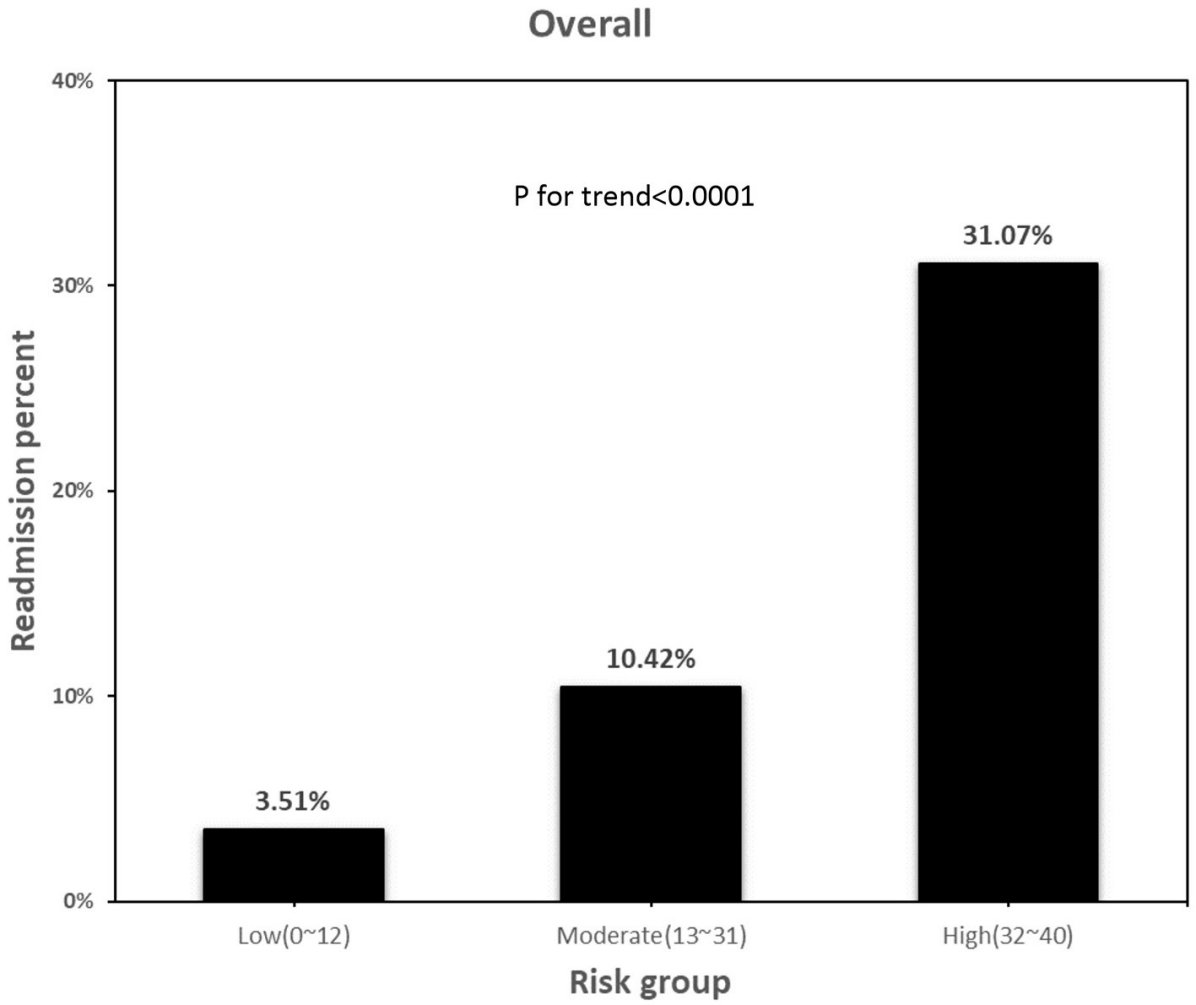
Readmission percentage in different risk group. Readmission percentage between overall cohort regarding low (0–12), moderate (13–31), high risk group (32–40). P for trend <0.0001 between 3 group.

The model had an AUROC of 0.7038 for predicting CCU readmission rate (Figure 3A). Validation studies of the model in the remaining 10% of our patients obtained an AUROC of 0.7181 (Figure 3B). Figure 4 compares the percentages of patients readmitted between overall cohort among the three groups. The percentage of readmitted patients significantly differed among the low-, moderate-, and high-risk groups (2.74, 10.2 and 31.73%, respectively; *p* < 0.0001). For predicting CCU readmission, our risk score model had a sensitivity of 54.51%, a specificity of 73.82% and a negative predictive value of 95.81%. The validation cohort had a sensitivity of 50.6%, a specificity of 73.67% and a negative predictive value of 95.76%. The validation study confirmed that the proposed risk prediction model had acceptable calibration, discrimination and validation.

## Discussion

To our knowledge, this study is the first to investigate risk factors for CCU readmission and to establish a model for predicting CCU readmission. This study revealed that readmission patients have a much higher mortality risk and a higher medical cost compared to non-readmission patients. The proposed model for predicting CCU readmission risk based on eleven known risk factors showed acceptable predictive accuracy in both the risk score model group and validation group.

### Special Findings of Our Study

As patients admitted to CCU have unique clinical characteristics, risk factors for readmission included cardiovascular medical history such as old age, CHF, peripheral artery occlusive disease, chronic kidney disease, diabetes mellitus, percutaneous coronary intervention, myocardial infarction and poor left ventricular ejection fraction. Additionally, sepsis and bleeding during CCU were associated with CCU readmission. Interestingly, patients who had received ECMO and patients initially admitted to CCU for in-hospital cardiac arrest had a lower readmission rate. We hypothesized that the lower readmission rate resulted from a higher mortality rate in these patients either during or after their CCU stay. These patients usually have poor prognosis and tend to be treated conservatively. After the patient is admitted to CCU, family members often realize that prognosis is poor and agree to sign a “do-not-resuscitate” order. Therefore, when the condition of the patient deteriorates, the patient is seldom transferred back to CCU. Additionally, certain demographic characteristics and comorbidities had strong associations with CCU readmission and poor outcomes. Readmission was significantly associated with long CCU stay, high mortality rate, and high medical costs.

### Comparison With Other Scoring Systems

Several studies in the literature have developed tools for predicting adverse outcomes after ICU discharge (4, 8–11). Most have assessed outcomes of readmission and hospital mortality in medical ICU and surgical ICU patients. For example, the Modified Early Warning Score developed by Reini et al. is often used to estimate the risk of ICU readmission within 72 h after ICU discharge (14). Gajic et al. (11) developed the Stability and Workload Index for Transfer score to predict ICU readmission within 7 days after ICU discharge. The calculated AUROCs ranged from 0.66 to 0.92 (4, 5, 11–14).

Mišić et al. (13) and Mišić et al. (12) also applied machine learning methods for readmission prediction among surgical populations (12, 13). Characteristics that increased the probability of CCU readmission in our study somewhat differed from those in previous reports. A common limitation of previous works is the lack of model calibration or discrimination and the lack of validation in CCU patients. Most CCU patients require care for an episode of acute CHF or acute myocardial infarction whereas most medical ICU patients are admitted for sepsis or acute respiratory failure (15, 16).

### Clinical Implications

This study developed and validated a model for using a comprehensive set of variables for clinical prediction of CCU readmission after discharge. After calibration, the proposed model demonstrated acceptable discrimination and calibration in identifying patients at high risk for CCU readmission at the time of CCU discharge. The model may be applicable for evaluating the efficacy of targeted therapeutic interventions aimed at reducing CCU readmission. Notably, none of the model parameters are modifiable in subsequent hospitalization. Therefore, to reduce the potential for CCU readmission, improved measures for preventing infection and hemorrhage should be implemented during the course of a CCU stay (17). To our knowledge, this model is the first designed specifically for predicting CCU readmission, and we hope that information provided by this model can be used to support clinical decision making, including discharge planning. Additionally, CCU physicians and personnel can use the model to determine whether a CCU patient can be safely discharged. Although not all CCU readmissions and deaths are avoidable, identifying high-risk patients can provide clinicians with insight into the appropriate timing of CCU discharge. For patients with high risk of CCU readmission or mortality, an appropriate CCU discharge plan should be made in advance and should include delayed discharge until stabilization, discharge to a step-down unit, and aggressive follow up in care wards, e. g., use of telemetry devices.

### Study Limitation

This study had several weaknesses inherent in the use of a database analysis. First, the analysis did not exclude patients readmitted to CCU for observation after a specialized procedure or operation, e.g., left main stent placement or carotid artery stenting. Second, due to resource allocation issues and lack of ICU beds, some patients designated for transfer to medical ICU were transferred to CCU instead. Third, since this study only included patients admitted to CCU, the prediction model developed in this study is not expected to be applicable to other care units such as ICUs. A final limitation is that, since this study analyzed data contained in a medical records database, the causes of CCU readmission could not be determined.

## Conclusions

In conclusion, this study identified 11 factors associated with increased CCU readmission risk and then developed a model for assessing this risk. The model may benefit clinicians in preventing CCU readmissions, which would then improve patient outcomes and medical care quality.

## Data Availability Statement

The raw data supporting the conclusions of this article will be made available by the authors, without undue reservation.

## Ethics Statement

The studies involving human participants were reviewed and approved by the Institutional Review Committee on Human Research at Chang Gung Memorial Hospital (IRB: 201900829B0C501). Written informed consent was not required for this study, in accordance with the local legislation and institutional requirements.

## Author Contributions

C-HT and C-IC conceived the idea. C-HT, T-YC, and P-JW implemented the study. C-CW, C-HL, W-JC, and C-IC supervised the study. C-HT and T-YC conducted data analysis. C-HT and P-JW interpreted the data. C-HT drafted the manuscript. C-IC provided critical revision. All authors reviewed the manuscript for important intellectual content, read, and approved the final manuscript.

## Conflict of Interest

The authors declare that the research was conducted in the absence of any commercial or financial relationships that could be construed as a potential conflict of interest.

## Publisher's Note

All claims expressed in this article are solely those of the authors and do not necessarily represent those of their affiliated organizations, or those of the publisher, the editors and the reviewers. Any product that may be evaluated in this article, or claim that may be made by its manufacturer, is not guaranteed or endorsed by the publisher.
