# Supplementary material for: Risk Stratification Model for Predicting Coronary Care Unit Readmission
Source: Front Cardiovasc Med. 2022 Feb 24;9:825181. doi: 10.3389/fcvm.2022.825181 (PMC8907527; doi:10.3389/fcvm.2022.825181)
Supplement: Supplementary file 1 [file Table_1.docx]

**Supplement Table 1 Predicted readmission rate by risk-cohort (validation)**

| **Risk score Cohorts** | **Score Total** | **Predicted risk of readmission** | **% of cohort**  **(n=2,778)** |
| --- | --- | --- | --- |
| Low risk of readmission | 0-12 | 0-5% | 1,632 (58.75%) |
| Moderate risk of readmission | 13-31 | 5-30% | 1,121 (40.35%) |
| High risk of readmission | 32-40 | >30% | 25 (0.90%) |
